# Inhibition continues to guide search under concurrent visual working memory load

**DOI:** 10.1167/jov.22.2.8

**Published:** 2022-02-14

**Authors:** Zachary Hamblin-Frohman, Stefanie I. Becker

**Affiliations:** 1 School of Psychology, The University of Queensland, Brisbane, Australia; 2 School of Psychology, The University of Queensland, Brisbane, Australia

**Keywords:** search, attention, inhibition, enhancement, suppression, VWM

## Abstract

It is well known that attention can be automatically attracted to salient items. However, recent studies show that it is possible to avoid distraction by a salient item (with a known feature), leading to facilitated search. This article tests a proposed mechanism for distractor inhibition: that a mental representation of the distractor feature held in visual working memory (VWM) allows attention to be guided away from the distractor. We tested this explanation by examining color-based inhibition in visual search for a shape target with and without VWM load. In Experiment 1 the presence of a distractor facilitated visual search under low and high VWM loads, as reflected in faster response times when the distractor was present (compared to absent), and in fewer eye movements to the salient distractor than the non-target items. However, the eye movement inhibition effect was noticeably weakened in the load conditions. Experiment 2 explored further, to distinguish between inhibition of the distractor color and activation of the (irrelevant) target color. Intermittently presenting single-color search trials that contained only either a target, distractor or a neutral-colored singleton revealed that the distractor color attracted attention less than the neutral color with and without VWM load. The target color, however, only attracted attention more than neutral colors under no load, whereas a VWM load completely eliminated this effect. This suggests that although VWM plays a role in guiding attention to the (irrelevant) target color, distractor-feature inhibition can operate independently.

## Introduction

Feature-based selection in visual search characterizes a set of mechanisms that allow an observer to bias attention toward specific objects that match certain properties or attributes. It has been hypothesized that attention is guided and biased toward items via an increase in sensitivity toward the defining feature of a sought-after object (i.e., target). For example, when a searcher's goal is to locate a blue item, sensitivity to blue is enhanced, and attention is guided first and foremost to matching blue items (Desimone & Duncan, 1995; Treisman & Gelade, 1980). However, this goal-driven, top-down mechanism of visual search competes with other stimulus-driven factors. Distractor items that are saliently different from others can supersede top-down strategies, causing attention to be directed to these irrelevant items (Theeuwes, 2004). Behaviorally, attentional capture by a distractor results in longer response times (RTs) to the target, or a large number of trials in which eye movements are first directed toward the distractor before they move to the target (e.g., Becker, 2010a; Theeuwes, Kramer, Hahn, & Irwin, 1998).

Although it has been proposed that salient distractor will always attract attention and the gaze (Itti & Koch, 2000; Theeuwes, 2004; for a review see Luck, Gaspelin, Folk, Remington, & Theeuwes, 2021), others have noted that salient distractors only capture when their features vary (e.g., when the color changes), and that these items can be ignored when their features are repeated over consecutive trials (Becker, 2008c; Becker, 2010a). Moreover, recent studies have shown that salient distractors can be actively inhibited, such that they are selected less frequently than other inconspicuous non-target items. For instance, in search for a shape-defined target (e.g., a blue diamond among varying blue non-target shapes), a repeated salient (e.g.) red distractor led to faster RTs, compared to distractor-absent trials, indicating that the presence of a distractor facilitated search (Gaspelin, Leonard, & Luck, 2015). Moreover, eye tracking measures revealed that the distractor attracted gaze less frequently than the other non-target items (Gaspelin, Leonard, & Luck, 2017).

Inhibition has previously been proposed as a mechanism that can increase search efficiency (Treisman & Sato, 1990). However, there are several conceivable mechanisms that could underlie inhibition. In the paradigm described above, inhibition could occur as a result of automatic carry-over effects that result from repetition of the distractor feature. Maljkovic and Nakayama (1994) were among the first to note priming effects with respect to the target stimulus: as the target feature repeated from trial to trial, search performance increased (i.e., leading to successively faster RTs). Becker (2007) showed that similar effects could be observed for a salient distractor; a salient distractor only captured attention and gaze when the color of the distractor and the other items swapped from the previous trial, whereas it could be successfully ignored when colors were repeated (Becker, 2007; Becker, 2010a). This implies that whatever capture effects the distractor originally had, its influence could be attenuated by repeated exposure.

In recent descriptions of visual search these types of priming effects have been collected under the umbrella term of selection history effects (Awh, Belopolsky, & Theeuwes, 2012; Failing & Theeuwes, 2018). Selection history effects are loosely defined as biases created from recent interactions with stimuli, it has been argued that these factors join top-down and bottom-up processes as a core mechanism that guides selection in the visual system (Wolfe, 2021). There are several varieties of selection history explanations for the mechanism responsible for distractor features inhibition. Intertrial priming, habituation, and statistical learning models have all been presented as explanations for distractor inhibition (Chelazzi, Marini, Pascucci, & Turatto, 2019; De Tommaso & Turatto, 2019; Ferrante, Patacca, Di Caro, Della Libera, Santandrea, & Chelazzi, 2018; van Moorselaar & Slagter, 2020). Critically, all of these proposals share the underlying premise that previous exposure to distractor properties leads to changes in behavior in subsequent search trials. The current work does not attempt to differentiate between these proposals and together are referred to as selection history effects throughout the rest of the article. In opposition to exposure-based explanations of feature inhibition, is the top-down–based, rejection template proposals.

It has been argued that inhibition is created, not as a result of selection history, but as a top-down mechanism designed to help guide attention to the target stimulus. Sawaki and Luck (2010) proposed in their *signal suppression hypothesis* that inhibition requires substantial top-down input and relies on working memory resources. It is well known that observers have top-down goals that help identify and locate target stimuli, possibly by biasing attention to the features stored in a mental representation of the target feature (“target template”; Duncan & Humphreys, 1989; Treisman & Gelade, 1980; Wolfe, 1994). Using this framework, inhibition could be described as the purposeful lowering of sensitivity toward the known distractor feature. This top-down suppression would ensure that items with the corresponding feature competes less for attention than other items, thus explaining how distractor presence facilitates visual search. It should be noted that proponents of rejection templates do not advocate that this form of inhibition is mutually exclusive with selection history explanations, but that both could work in conjunction (Geng, Won, & Carlisle, 2019).

There are substantial links between working memory and the ability to control attentional search, providing inferential support for this proposition. For example, individuals with high working memory capacity are better able to resist attentional capture by singletons than individuals with a lower capacity (Fukuda & Vogel, 2009). Moreover, singleton capture increases when working memory is loaded (Burnham, Sabia, & Langan, 2014; Lavie & De Fockert, 2005), and inhibition of spatial locations seems to be linked to spatial working memory (Dube, Basciano, Emrich, & Al-Aidroos, 2016). These findings render it quite plausible that a top-down template is responsible for inhibiting known distractor features and help guide attention toward the target stimulus.

Arita, Carlisle, and Woodman (2013) argued that a *template for rejection* can be created by storing distractor feature information in visual working memory (VWM), in the form of a distractor template that in turn serves to inhibit upcoming distractors on a given trial. To test this hypothesis, the authors used a cuing paradigm. Before a visual search array, participants were provided with a positive (indicative of the target feature), negative (the distractor feature), or neutral cue (unrelated to the upcoming search). They found RT benefits for both positive and negative cues in comparison to neutral cues. Because exposure was limited to just the cue information, item selection history could not account for these results and, correspondingly, Arita and colleagues (2013) claimed that the results provided evidence of working memory–based inhibition. However, a potential limitation of the study was that it used visual search arrays that split the stimulus features into separate visual fields. For example, on the left were all distractor stimuli and on the right potential targets. In subsequent studies the negative cuing effect was only observed when potential targets and distractors were spatially separated in this way (see Becker, Hemsteger, & Peltier, 2015). When target and distractor features were intermixed, no benefit of negative cues was observed, implying that in the original paradigm participants were able to use a spatial elimination strategy to rapidly disengage attention from the distractor hemi-field and switch to the potential targets. However, a more recent study by Carlisle and Nitka (2019) was able to replicate the RT facilitation effect even when potential targets and distractor were intemixed in the search arrays, seemingly in direct cotntradiction to the previous work (Becker et al., 2015).

Multiple studies have used similar paradigms and have shown conflicting results regarding distractor templates. Kawashima and Matsumoto (2018) observed behavioral costs of negative cues at all stages of search, whereas Zhang, Gaspelin, and Carlisle (2020) found that negative cues led to search facilitation only when they were presented for a sufficiently long duration. Beck, Luck, and Hollingworth (2018) found that attention was captured by memory-matching items (including from negative cues) but that this information was later used to avoid known irrelevant features. Because there is such a large discrepancy in the behavioral findings of the negative cuing paradigm, it is difficult to draw conclusion from these results. It is possible that, depending on the paradigm, participants were more or less encouraged to create rejection-templates whereas in others this process was eliminated. In addition, there could be another mechanism (such as the spatial elimination strategy) that can account for the negative cue benefit observed in a subset of previous studies. Research encorporating neural measures are similarly conflicting. With arguments being made both for (e.g., Reeder, Olivers & Pollmann, 2017; but see Reeder, Olivers, Hanke, & Pollmann, 2018) and against (e.g., Berggren & Eimer, 2021) the existance of active negative templates. Again the negative cuing paradigm led to contradictory behavioral results and inconclusive evidence regarding the presence of rejection-based distractor templates.

Even if observers are unable to create a VWM rejection template on a trial by trial basis in the cuing paradigm, this does not ensure that inhibition is completely independent of memory. The task of the negative-cuing paradigm is quite complex: participants must have variable templates for cues being indicative of the target or distractor (or neutral), as well as keeping track of the colors of the stimuli that vary from trial to trial. It is possible that inhibition is unable to take effect under these scenarios, or that it is too weak (or imprecise) to produce a tangible or consistent effect. Potentially an inhibitory template needs the to-be ignored item to be attended to incorporate its feature information into the visual system to allow for future strategic behaviors. When distractor-feature consistency is retained (i.e., when the distractor feature is repeated), inhibition effects can be seen as early as trial *n* + 1 (Gaspelin, Gaspar, & Luck, 2019), which is consistent with this explanation (but also an selection history explanations).

The idea that to-be-suppressed contents may need to be attended before they can be effectively used for inhibition is reminiscent of Ironic Process Theory (Wegner, 2009), which details how the act of suppressing specific thoughts can make them more likely to emerge. This has been extended to visual search. Huffman, Rajsic, and Pratt (2019) showed that when participants were explicitly informed of the distractor's location prior to search, capture by the distractor was more likely than when no information was given. These effects could also apply to the previous negative cuing studies. In standard inhibition studies, the core goal for the participant is to locate the target item (the same as a positive cue trial), while ignoring the distractor could be considered a secondary goal. In the cuing studies, when a negative “rejection” cue is given, the task priority may switch to, first, ignoring the distractor feature and *then*, second, locating the target item. This active prioritization of the distractor item may render it prominent, not just in working memory, but within consciously operating systems. This conscious “rejection” of the distractor color may in fact lead to its later selection in subsequent search and explain the RT costs of the negative cues. Taking this into consideration, a working memory explanation of inhibition may still be viable (with some alterations).

Although studies suggesting that top-down systems are unable to inhibit a cued novel distractor feature have returned inconclusive results, it is also uncertain whether static feature inhibition is due to a template-related mechanism or emerges through exposure-based effects. The key concern is that in inhibition designs it appears impossible to disentangle feature-suppression from selection history effects when the distractor feature is held constant. As soon as trial *n* + 1, suppression of a repeated distractor feature can be accounted by either a template or selection history explanations. However, although selection history and inhibition will likely always co-vary, working memory and inhibition can be disentangled.

In the present study, we tested whether inhibition relies on working memory resources by varying VWM over visual search trials. If inhibition is dependent on VWM resources, it should be reduced or eliminated under load. VWM has a strict capacity limitation, meaning that different processes compete for prioritization (Kiyonaga & Egner, 2013). By adding VWM load to the visual search trials we should be able to create conditions where visual search is completed with or without available working memory resources. Inhibition was measured by assessing the mean RT to the target in the presence versus absence of an irrelevant distractor that repeated in color. Moreover, to ensure that RT benefits on distractor present trials indeed reflect early, attentional processes and not decisional or response-related processes, we assessed the proportion of first eye movements in a trial directed to each stimulus type. Inhibition of the distractor should result in less frequent selection of the distractor, compared with the other, inconspicuous non-target items (e.g., Gaspelin et al., 2019). In Experiment 2 we extend this paradigm by exploring whether a VWM load differentially impacts search for the target feature or the inhibition of the distractor feature.

## Experiment 1

Experiment 1 used a commonly used inhibition paradigm (Gaspelin et al., 2015); a visual search task for a specific shape target among a heterogeneous background (i.e., non-targets of different shapes, but all the same color as the target). On 50% of trials, one of the non-targets was replaced by a distractor with a color that remained consistent through the experiment. To assess whether working memory is a requirement for a distractor feature to be suppressed, we varied the memory load between blocks. Visual search trials were completed with either a no-load, a two-memory, or a four-memory load task.

If working memory resources are required to maintain an inhibitory template, then inhibition of the distractor should be reduced in the low load condition and even more strongly reduced (or absent) in the high load condition. If, however, inhibition does not rely on working memory resources, then suppression effects should be seen regardless of concurrent memory load. Inhibition was assessed both by using the “standard” measure of mean RT to the target and the proportions of first eye movements to target, non-targets, and the distractor. Successful inhibition of the distractor should result in shorter RTs when the distractor is present versus absent and fewer eye movements to the distractor than any of the inconspicuous non-targets.

## Methods

### Participants

Twenty-eight first-year participants (*M* age = 21.3, 19 female) from the University of Queensland participated in the experiment. The BUCSS tool (Anderson, Kelley, & Maxwell, 2017) was used to estimate sample size. The oculomotor suppression effect of the distractor compared to non-target items (*t*(47) = 5.35) observed in a previous eye tracking study (Hamblin-Frohman, Chang, Egeth & Becker, *submitted*) was used for the estimate. To achieve an estimated power of 90% (with 75% assurance) the BUCSS tool suggested a sample of 27. Three participants were excluded for consistently (>50% of trials) beginning eye movements before the presentation of the search array in the load conditions, leaving 25 participants for the final analysis. The estimated sample size (using the same parameters) for 85% power was 23 participants; thus additional data were not collected after the exclusions were made. All participants reported normal or corrected-to-normal vision. Study approval was granted by the University of Queensland's School of Psychology Ethics Board.

### Apparatus

Stimuli were presented on a 21-inch CRT monitor (refresh: 60 Hz). A chin and head-rest was used to hold the participant's heads in a constant position 600 mm from the screen. Gaze location was measured by an SR-Research Eyelink-1000 eye tracker at 500 Hz sampling rate. The experiment was controlled by Python's PsychoPy (Peirce, 2007).

### Stimuli

Stimuli were presented against a white background. Throughout all trials a fixation cross was drawn at the center of the screen. The search stimuli were presented in a diamond configuration, with each being 6.68° visual angle away from the fixation cross. The search stimuli consisted of four different shapes: the target diamond (1.43° × 1.43°), and a square (1.43° × 1.43°), circle (diameter: 1.72°) and hexagon (height: 2.00°, width: 1.43°) as non-target shapes. Within each of the search shapes we presented an arrowhead (“<” or “>”) as a response-defining item (length: 0.6°).

The memory stimuli were colored squares (1.43° × 1.43°) also presented in a diamond configuration (the top and bottom memory items were removed in the low-load conditions) but closer to fixation (2.5° away). This eliminated the need to make eye movements in the memory encoding phase and ensured there was no spatial overlap between search and memory stimuli. Seven equiluminant (30 ± 2 cd/m^2^) colors were used in the experiment: red, gold, green, blue, orange, purple, and teal. Each participant was randomly assigned one color as the color of the target and non-targets and another as the distractor color. The other colors were used for the memory array, whereby the colors used in the memory arrays never appeared in the search trials. The colors used in the memory and visual search tasks were kept separate to allow optimal conditions for inhibition of the distractor color (Carlisle & Kristjánsson, 2018) and prevented memory-driven attentional capture effects (Olivers, Meijer & Theeuwes, 2006). Participants were not given specific instructions to move their eyes; however, the search items were far from fixation and contained small characters, encouraging eye movements.

### Design and procedure

The three load conditions were presented as three (counterbalanced) blocks, each containing 120 trials. At the beginning of the experiment, participants completed 40 practice search trials under no VWM load. Before the first load block began, participants completed eight practice trials to learn the procedure.

Each trial (in the load conditions) consisted of two tasks: a visual search and change detection task. The visual search task required participants to respond with a left or right arrow key corresponding to the direction of the arrowhead contained within the target (the diamond shape). On 50% of trials one of the non-target items was changed to have the distractor color. Participants were informed that this would never be the target. The change detection task required participants to remember the colors of two (low load) or four (high load) squares. On 50% of trials one of the colors changed and on the other 50% there was no change. Participants were required to respond with the “s” key if they thought all colors were the same, or the “d” key if one was different.

In the no load block each trial began with participants maintaining fixation for 500 ms, after which the search array was presented. The search stimuli were displayed until a response was made; however, if the participant made a response later than 1500 ms, a feedback message was displayed reading “Too Slow!”

In the low-load and high-load conditions, the search trials were bookended by a color change-detection task. After fixation, a memory array was presented for 500 ms; then after a blank screen lasting 500 ms, the search array was presented (for the same duration as the no-load condition), which was presented until the participant responded. After a search response was recorded, the memory array returned (locked to 2000 ms after the offset of the first memory array), and participants made the change detection judgment. If the response to the search array was slower than 1500 ms, the trial was cancelled, and the memory test did not occur. This ensured that participants still prioritized performance in the visual search section of the task and that memory retention times were the same across conditions.

## Results

### Response times

Trials were excluded if RTs were longer than 1500 ms (5.8%) or if an incorrect search response was recorded (6.5%). A 2 (Distractor: Present, Absent) × 3 (Load: None, Low, High) repeated measures analysis of variance (ANOVA) was conducted on RT data. The effect of Distractor presence was significant *F*(1, 24) = 59.85, *p* < 0.001, ƞ^2^_p_ = 0.71, indicating that RTs were shorter when the distractor was present compared to absent (see Figure 2). A significant effect of Load was also present, *F*(2, 48) = 5.90, *p* = 0.005, ƞ^2^_p_ = 0.20. Indicating that RTs in the no load condition was shorter than the low or high loads). This appears to be a generalized interference on search performance due to memory load (see Woodman & Luck, 2004), as the Distractor x Load interaction was not significant *F*(2, 48) = 2.16, *p* = .126. This indicated that the RT facilitation observed in the distractor-present trials was consistent across load conditions and hence, that inhibition continued to facilitate search under VWM load.

### Eye movements on distractor-present trials

Trials were excluded (6.5%) from all eye movement analyses if saccades were initiated too early (100 ms) or too late (1000 ms). To assess if the effects obtained in the mean RTs reflected early, attention-guiding processes, a 3 (Search Item: Target, Non-Target, Distractor) × 3 (Load: None, Low, High) repeated measures ANOVA was conducted on proportions of first eye movements on distractor-present trials (depicted in Figure 3). The probability of selecting a non-target was computed as the total amount of non-target first fixations divided by the amount of non-target in that display type. The results showed a significant main effect of search item, *F*(2, 48) = 177.20, *p* < 0.001, ƞ^2^_p_ = 0.88, indicating that the target attracted more first eye movements than the average non-target. Importantly, non-targets also received a higher proportion of first eye movements than the salient distractor, reflecting inhibition of the distractor (see Figure 3). The effect of load was non-significant *F*(2, 48) = 0.50, *p* = 0.608. Interestingly, the item × load interaction was significant, *F*(4, 96) = 11.08, *p* < 0.001, ƞ^2^_p_ = 0.32. Pairwise two-tailed *t*-tests revealed that the inhibition effect was significant across all load conditions: The distractor item consistently received a lower proportion of first eye movements than the non-targets, in the no load: *t*(24) = 8.18, *p* < 0.001, 95% confidence interval (CI) [9.7, 16.3], low-load: *t*(24) = 3.91, *p* = 0.003, 95% CI [1.8, 7.8] and high-load conditions: *t*(24) = 3.00, *p* = 0.006, 95% CI [1.6, 8.6]. However, there were significantly lower proportions of distractor capture in the no-load compared to the low-load condition, *t*(24) = 5.04, *p* < 0.001, 95% CI [4.3, 11.2], and high load condition, *t*(24) = 5.82, *p* < 0.001, 95% CI [5.2, 11.0]. Distractor fixations did not differ between the low and high memory loads, *t*(24) = .20, *p* = 0.841, 95% CI [−2.7, 3.3].

### Eye movements to the target

To assess if the presence of a distractor benefited target selection, we computed a 2(Distractor: Present, Absent) × 3(Load: None, Low, High) ANOVA on the proportion of first fixations directed toward the target. In the presence of the distractor, the search target was more likely to be selected with the first eye movement (*M* = 39.6%) than when the distractor was absent (*M* = 36.5%): *F*(1, 24) = 9.40, *p* = 0.005, ƞ^2^_p_ = 0.28. When working memory load was present, target selection rates decreased (*M*_Low_ = 36.4%, *M*_High_ = 35.5%) compared to the no load condition (*M* = 42.2%): *F*(2, 48) = 8.61, *p* = 0.001, ƞ^2^_p_ = 0.26. There was no interaction between these effects *F*(2, 48) = 0.17, *p* = 0.844, implying that the benefit of distractor presence continued to aid target localization under all load conditions, and that there was an overall impact of memory load on target localizations independent of distractor presence.

### Change detection performance

As a manipulation check, we assessed accuracy in the memory task. Memory accuracy was significantly higher in the low load condition (*M* = 85.0%) than in the high load condition (*M* = 66.8%), *t*(24) = 12.68, *p* < 0.001, 95% CI [15.2, 21.2], indicating that our manipulation of load was successful. Furthermore, one-sample *t*-tests revealed that both low- and high-load conditions performed significantly above chance level (50%) and below ceiling (100%), all *p*s < 0.001, indicating that the participants were successfully completing the memory task even in the high load condition. Memory performance did not differ as a function of distractor presence for either load condition, both *p*s > 0.415, indicating that change detection accuracy was not influenced by distractor presence. Further analysis of the relationship between memory performance and inhibition is explored in the Appendix.

## Discussion

Experiment 1 tested whether inhibition of a distractor color could guide search when VWM was taxed by a concurrent memory task. For the mean RTs, the distractor presence led to faster search under all load conditions. This indicated that inhibition of the distractor did not require working memory resources, as the facilitation benefit remained consistent across no, low and high memory loads. However, the first eye movement data revealed that search behavior was influenced by memory. Under load, the distractor was still less likely to attract the first eye movement than the non-target items; however, the effect was reduced in comparison to the no-load condition. Interestingly the low- and high-load conditions did not differ at all in this regard, suggesting that reduced inhibition was not due to the amount of available memory resources but perhaps to general interference from the dual-task design. Although this explanation would render the results consistent with inhibition being independent of working memory, the discrepancy needs to be accounted for to safeguard this conclusion.

A few explanations could account for the weakening of inhibition effect under load. First, this may have been simply due to generic interference from the dual-task paradigm. Adding memory load can hinder visual search (Woodman & Luck, 2004; Woodman, Vogel, & Luck, 2001), potentially adding an extra layer of noise into the search data. This is observed in the RT data: although the influence of the distractor did not change with load, adding working memory load led to overall slower responses. Furthermore, target capture rates were also impacted by memory load, indicating that VWM load interfered with general search performance. However, the increase in first fixations to the distractor item under load is still unaccounted for because a corresponding increase in non-target first fixations was not observed.

A second possibility was that the manipulations of working memory load were too strong. It is possible that memory resources were completely depleted even in the low-load memory condition. Combined with concurrent task information (search for the diamond, make a keyboard response, ignore *x* color), the memory system may have been overloaded. This would explain the lack of difference between the low and high load conditions. However, as we still observed significant inhibition across the load conditions, this would imply that feature-suppression does not completely rely on working memory resources. Furthermore, memory accuracy differences were observed between low and high loads, suggesting that the high load condition required significantly more working memory resources than the low load condition. Taken together, these findings render it unlikely that working memory resources were fully depleted in the low load condition.

**Figure 1.**
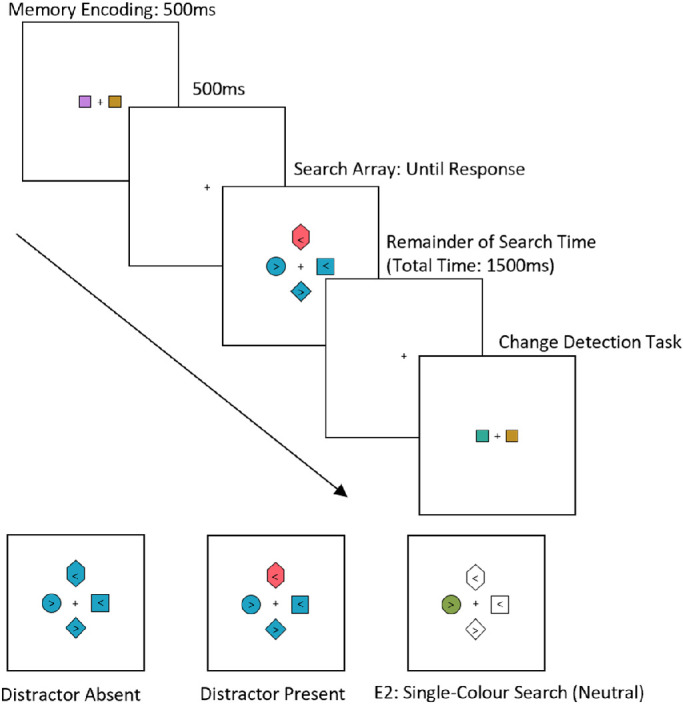
*Above:* Example of a trial in the low-load condition. After fixation, a memory array was presented briefly that contained two colored squares in the low-load condition or four colored squares in the high load condition. Participants then completed a visual search task for the diamond target and responded to the direction of the *arrowhead* inside. After the response to the search task, the memory array reappeared (always 2000 ms after the offset of the first memory array), and participants reported whether the display was the same or whether one of the squares had changed (50% of trials). In the no load condition only the visual search portion of the trial was presented. *Below:* The types of visual search trials; 50% of trials were distractor present trials, and 50% absent trials. In Experiment 2, 30% of trials were *single-color* search trials with only one non-target item colored as the target, distractor or neutral (depicted), rendering it the color singleton. Participants continued to search for the target-diamond shape following the same procedure as the normal visual search trials.

A third explanation derives from more recent findings suggesting that the inhibition effect does not solely consist of distractor-feature suppression, but also target-feature enhancement (Chang & Egeth, 2019; Hamblin-Frohman et al., *in revision*). In the present visual search task, observers may not only attend to the shape of the target, but also to its color (e.g., blue). Even though the target and non-targets have the same color, biasing attention to the target color could enhance target selection by guiding selection away from the distractor color (e.g., red; see Figure 1^2^,^3^). In distractor-present trials the RT facilitation could then be explained by reducing the set-size of the attended items. Chang and Egeth (2019) found support for both target enhancement and distractor inhibition influencing visual selection. In their study, they intermixed *probe trials* with the visual search task, allowing separate assessment of target and distractor colors by separately comparing attentional allocations toward them compared to neutral colors. The results showed a significant benefit for target colored probes over neutral probes, indicating target enhancement, and significant impairments in responding to distractor colored probes (compared to neutral probes) reflecting inhibition of the distractor color. Hamblin-Frohman and colleagues (*submitted*) extended the methods by assessing eye movements on probe trials and confirmed that both target enhancement and distractor suppression affected early attention-guiding processes.

**Figure 2.**
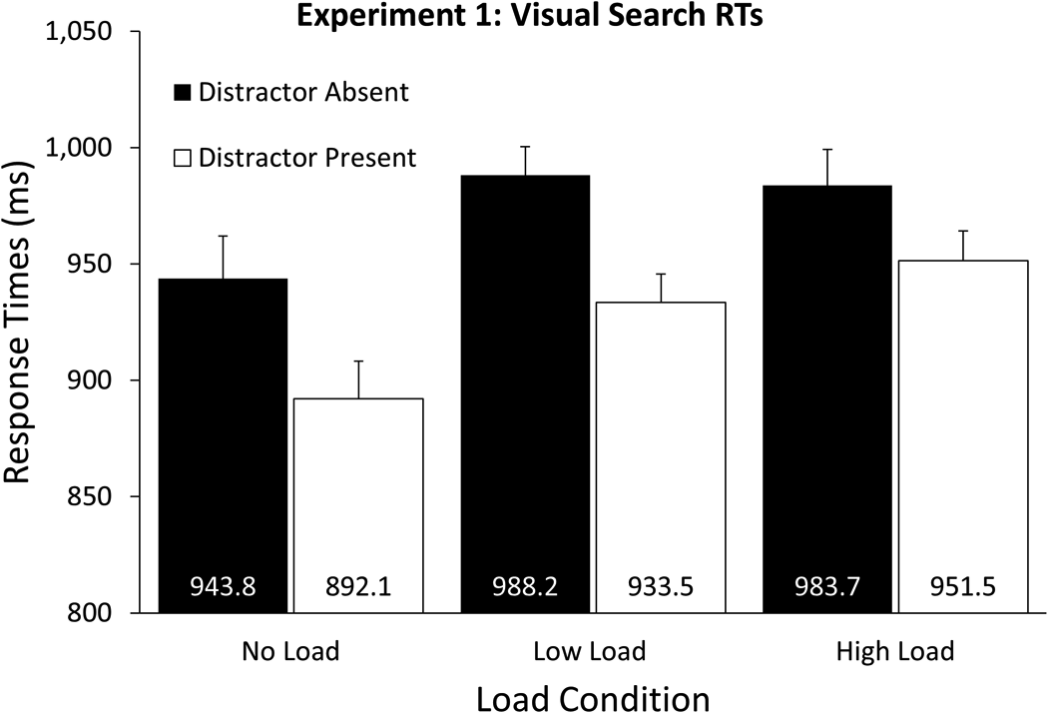
Response Time results from Experiment 1, depicted separately for the different load conditions and distractor present versus absent trials. Benefits of distractor presence were observed across all conditions, because RTs were faster when the distractor was present than when it was absent. Memory load also influenced response speed, such that RTs were shorter in the no-memory load condition than the low or high loads. Importantly, the benefits from distractor presence were consistent across all load conditions. *Error bars* represent within-subject 95% CIs (Loftus & Masson, 1994).

**Figure 3.**
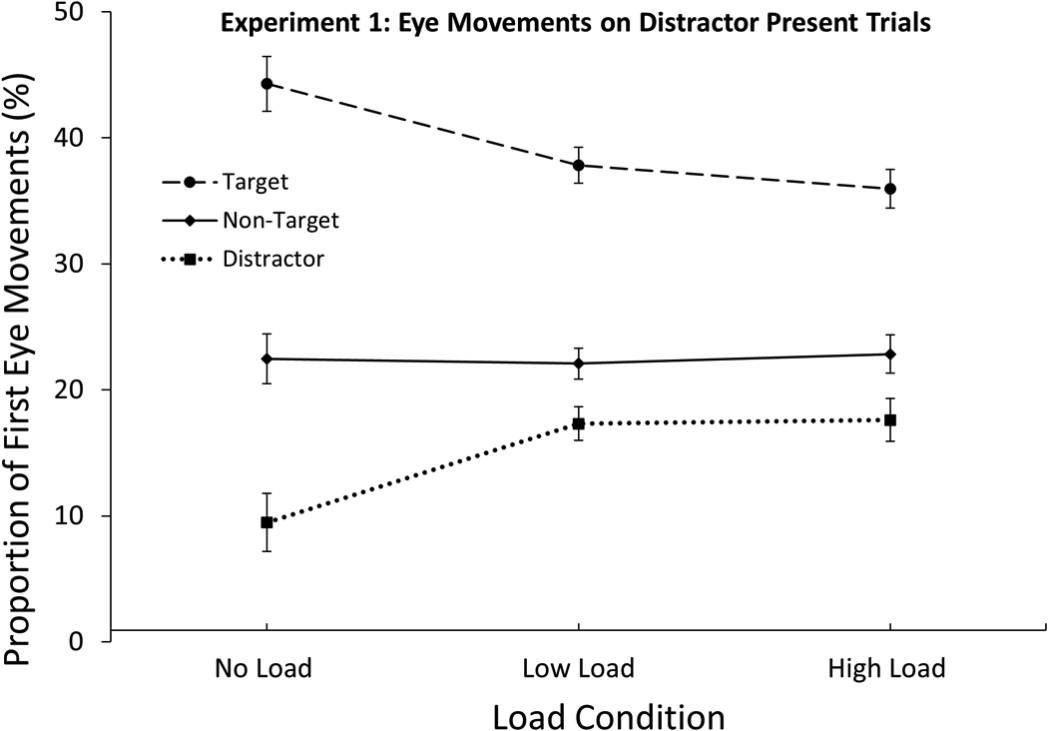
Proportions of first eye movements directed toward the different stimuli on *distractor-present* trials. The distractor consistently received less first eye movements than the other non-target items. However, this effect was weaker in the load conditions than under no load. *Error bars* represent within-subject 95% CIs (Loftus & Masson, 1994).

These results demonstrated that both target-feature enhancement and distractor-feature suppression can be used to guide search to the target. Critically, in the present study it was likely that the suppression effect observed under no memory load was the product of both target-feature enhancement and distractor-feature suppression. The attentional template that guides attention is widely regarded to be stored in working memory (Desimone & Duncan, 1995; Olivers, Peters, Houtkamp, & Roelfsema, 2011). Thus, when memory load was added this may have blocked target-feature enhancement, leaving only distractor-feature suppression to guide attention away from the distractor item, accounting for the lessening of the oculomotor suppression effect. It is possible that only distractor-feature suppression was operating under load whereas target enhancement was eradicated, explaining the weakened effect. Experiment 2 was designed to address this question by disentangling target enhancement and distractor suppression under VWM load.

## Experiment 2

The aim of Experiment 2 was to distinguish between two possible mechanisms of target localization benefits—target feature enhancement versus distractor feature suppression—and assess whether either one depended on working memory resources. To that aim we included *single-color* *search* trials in the visual search task of Experiment 2. Single-color trials were identical to the normal search trials, except that the stimuli were rendered with no fill color. Only one of the non-target shapes was colored, the singleton item could have the color of the target, distractor, or a neutral color (one not used in the search task). The task was the same as the visual search trials (i.e., participants had to locate the target shape and respond to the small arrowhead inside the target). Presenting each of the colors (target, distractor, neutral) in isolation and assessing eye movements to the different colored singletons allowed assessment of the attentional biases to the search-related features in comparison to a neutral feature.

Under no VWM load the individual effects of target-feature enhancement and distractor-feature suppression should be observed. There should be an increase in attentional bias to the target-colored singleton, and a decrease in selectivity for the distractor-colored singleton (both drawn in comparison to the neutral colored singleton). Critically, when load is added to the single-color search trials, its effect on both target-feature enhancement and distractor-feature suppression can be observed. If target-feature enhancement is impacted by load, then it should display no more bias than the neutral color. Conversely, if the distractor singleton no longer shows a reduced attentional bias compared to the neutral singleton, then this will be evidence that distractor feature suppression is attenuated by load. If, however, both the target singleton and distractor singleton continue to display attentional bias effects (albeit to a weaker amount), then this would suggest that VWM load simply impaired visual search due to the additional dual task costs.

## Methods

### Participants

Thirty-eight first-year participants (*M* age = 20.3, 29 female) at the University of Queensland participated in the experiment. Using the effect size from the low load, oculomotor suppression effect in Experiment 1 (*t*(24) = 3.91) and the same power parameters (desired power: 90%; assurance: 75%) the BUCSS tool suggested a sample size of 34 (Anderson et al., 2017). Three participants were excluded for making eye movements before the onset of the search array (>50% of trials). One participant was excluded for using a spatial search strategy (first fixation directed to the same location on >80% of trials). This left 34 participants for the analyses. All participants reported normal or corrected to normal vision, and study approval was granted by The University of Queensland's School of Psychology Ethics Board.

### Apparatus and stimulus

Experiment 2 used the same equipment and stimuli as Experiment 1. As there were no differences between low and high memory loads and the inclusion of the rare single-color search trials, the high load condition was not included in Experiment 2. On the new *single-color* search trials the items were rendered only as a black outline with no fill color (see Figure 1). One of the non-target items was rendered as the target color, the distractor color or as an unrelated color (becoming the singleton item) that changed on each presentation (this was never one of the memory colors of that trial in the load condition).

### Design and procedure

Trial structure and timings were the same as Experiment 1, with the distractor appearing on 50% of the normal search trials (referred to as *visual search trials* in the analysis), and the memory task bookending each search in the load condition. Thirty percent of trials were the new *single-color* *search* trials. Participants continued to search for the diamond-target, which was always just the shape outline. One of the non-target items became a singleton, colored either as the target, distractor, or neutral color that varied on each appearance (10% of trials each). Participants again completed a warm-up block (40 trials). Each load block (counterbalanced) contained 240 trials (with 80 being single-feature search trials), for a total of 480 experimental trials.

## Results

Trials were excluded if RTs were longer than 1500ms (7.2%) or if an incorrect search response was recorded (5.5%) over both trial types. Trials were excluded (13.1%) from all eye movement analyses if saccades were initiated too early (100 ms) or too late (1000 ms). A single-sample *t*-test revealed that change detection accuracy (89.3%) was significantly lower than ceiling performance (100%), *t*(34) = 8.69, *p* < 0.001.

### Visual search: Response times

**Figure 4.**
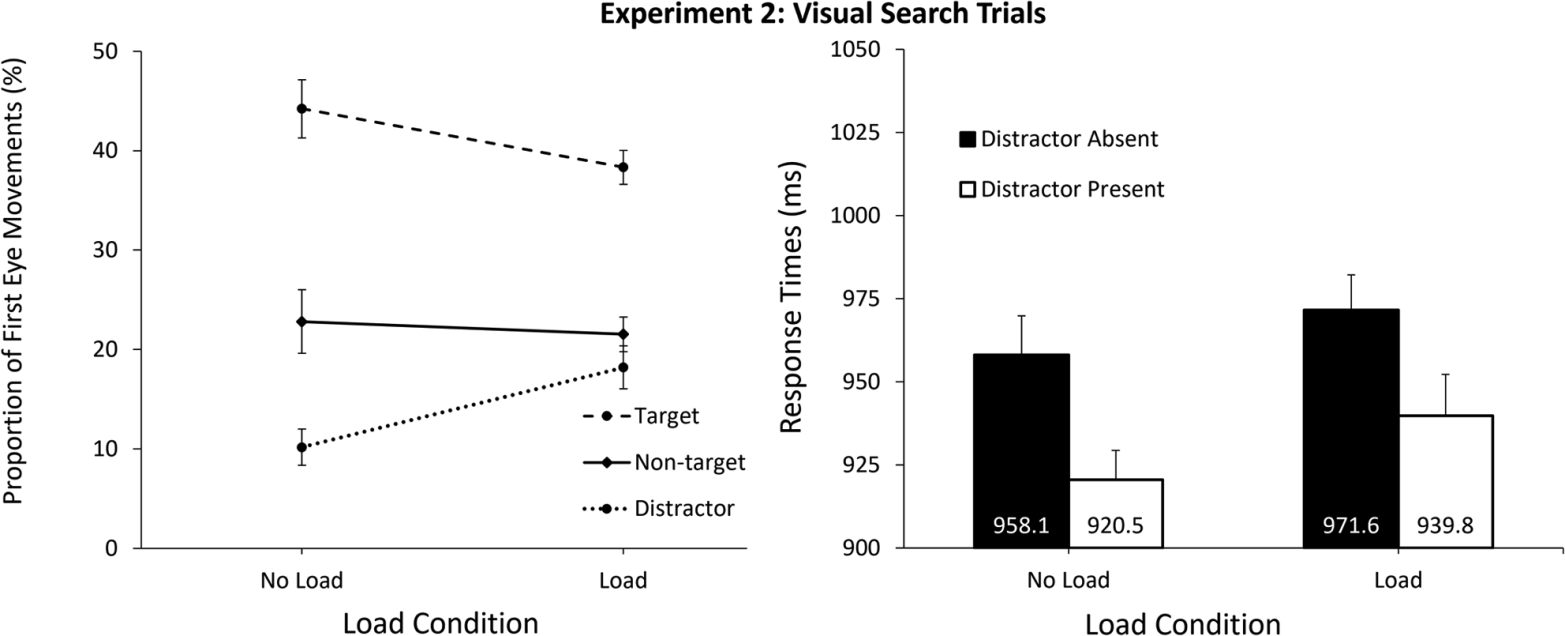
Data for the visual search trials in Experiment 2. *Left:* Eye movements on distractor-present trials across both load conditions. Results revealed that there were more first eye movements directed to the distractor under load compared to without; however, in both conditions capture was lower than the average non-target item. *Right:* RTs for distractor absent versus present trials across load conditions. Results display the RT facilitation of distractor presence in both load conditions. Although RTs were overall slower under load, there was not a significant difference compared with the no-load condition, differing from Experiment 1. *Error bars* represent within-subject 95% CIs (Loftus & Masson, 1994).

The visual search results of Experiment 2 were similar to the results of Experiment 1. A 2 (Distractor: Present, Absent) × 2 (Load: No load, Load) repeated measures ANOVA was conducted on the mean RTs in the visual search trials (see Figure 4). As in Experiment 1, a significant effect of distractor presence was observed, *F*(1, 33) = 36.73, *p* < 0.001, ƞ^2^_p_ = 0.53, with RTs being faster on distractor present compared to absent trials. However, differing from Experiment 1, RTs did not significantly differ between load conditions, *F*(1, 33) = 2.39, *p* = 0.132. Once again there was no interaction between load and distractor presence for mean RTs, *F*(1, 33) = 0.33, *p* = 0.569.

### Visual search: Eye movements on distractor-present trials

The eye movement data more closely resembled the results of Experiment 1. A 3 (Search Item: Target, Non-Target, Distractor) × 2 (Load: No load, Load) repeated-measures ANOVA was conducted on proportions of first eye movement locations on distractor-present trials (see Figure 4). The effect of item type was significant, *F*(2, 66) = 100.54, *p* < 0.001. ƞ^2^_p_ = 0.75, indicating that the target received more first eye movements than the non-targets, *t*(33) = 8.73, *p* < 0.001, and that the non-targets received more first eye movements than the distractor, *t*(33) = 8.28, *p* < 0.001. There was no effect of load condition, *F*(1, 33) = 0.71, *p* = 0.505. Importantly the interaction between load and item type was significant, *F*(2, 66) = 21.79, *p* < 0.001. ƞ^2^_p_ = 0.40. The color distractor received less first eye movements compared to the average non-target item in the no load condition, *t*(33) = 10.06, *p* < 0.001, 95% CI [9.9, 15.0] and the load condition, *t*(33) = 2.33, *p* = 0.026, 95% CI [0.4, 6.0], revealing that the distractor color was inhibited under load. Moreover, the data from the first eye movements showed significantly more eye movements to the distractor in the load condition, compared to the no load condition, *t*(33) = 6.78, *p* < 0.001, 95% CI [5.6, 10.4], corresponding to the results of Experiment 1.

### Single-color search: Response times

**Figure 5.**
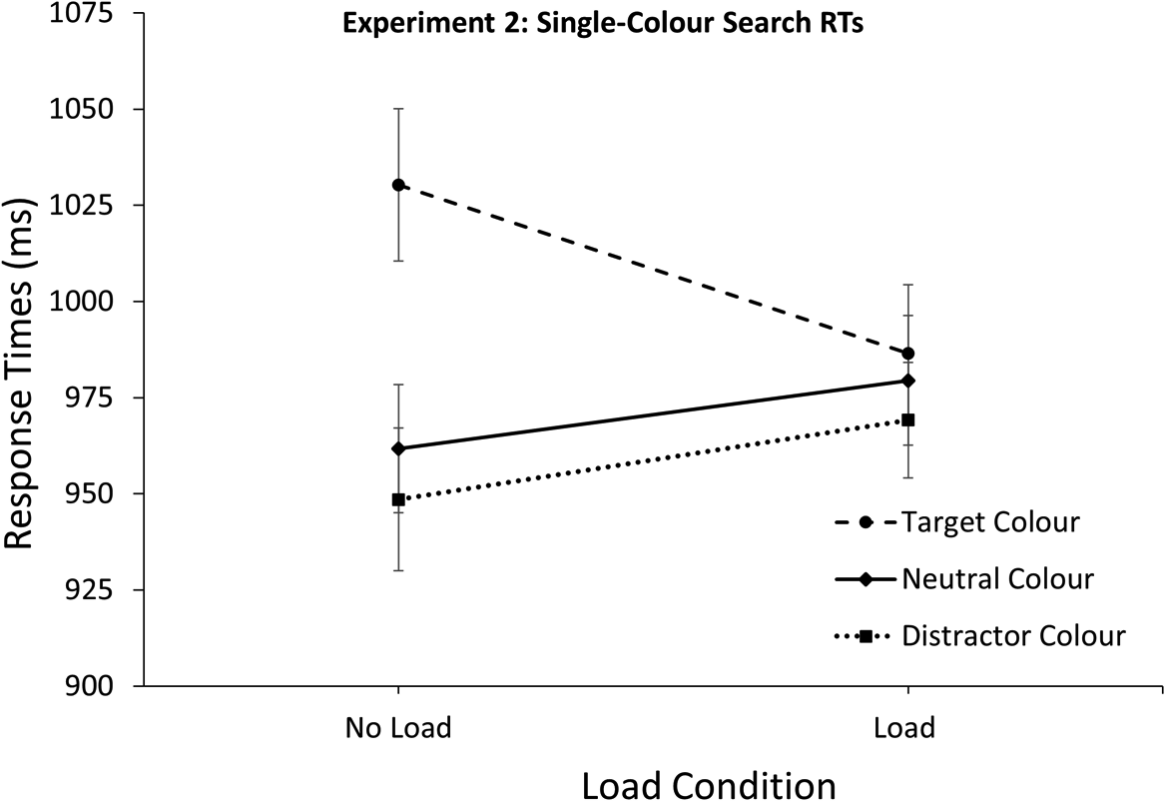
Response times for the single-color search trials in Experiment 2. Results revealed no significant differences between the presence of a distractor colored singleton and a neutral colored singleton. The target color led to slower RTs under no load but interfered less with the addition of memory load. *Error bars* represent within-subject 95% CIs (Loftus & Masson, 1994).

The mean RT for the single-color trials are depicted in Figure 5. A 3 (Singleton Color: Target, Distractor, Neutral) × 2 (Load: No load, Load) repeated measures ANOVA computed over the mean RTs of the single-color trials showed no significant effect of load, *F*(1, 33) = 0.14, *p* = 0.905, but a significant effect of color, *F*(2, 66) = 18.21, *p* < 0.001, ƞ^2^_p_ = 0.36. Importantly, these effects were qualified by a significant interaction *F*(2, 66) = 8.94, *p* < 0.001, ƞ^2^_p_ = 0.21. Without memory load, color had a significant effect on RTs, *F*(2, 66) = 21.45, *p* < 0.001, ƞ^2^_p_ = 0.39, with the target-colored singleton leading to slower RTs compared to the neutral colors *t*(33) = 5.42, *p* < 0.001, 95% CI [42.8, 94.3]. However, no difference between the neutral and distractor color singleton was observed, *t*(33) = 1.07, *p* = 0.291, 95% CI [-38.2, 11.8]. Under memory load the different singleton types no longer influenced RTs, *F*(2, 66) = 1.32, *p* = 0.270, revealing that the distracting effect of the target-color singleton was no longer present under load. Comparing the mean RTs across load condition for target-color singletons revealed that responses were in fact *faster* under load than without load, *t*(33) = 2.22, *p* = 0.033, 95% CI [3.8, 83.7], indicating that working memory load reduced the contingent capture effects of the target-colored singleton.

### Single-color search: Eye movements

**Figure 6.**
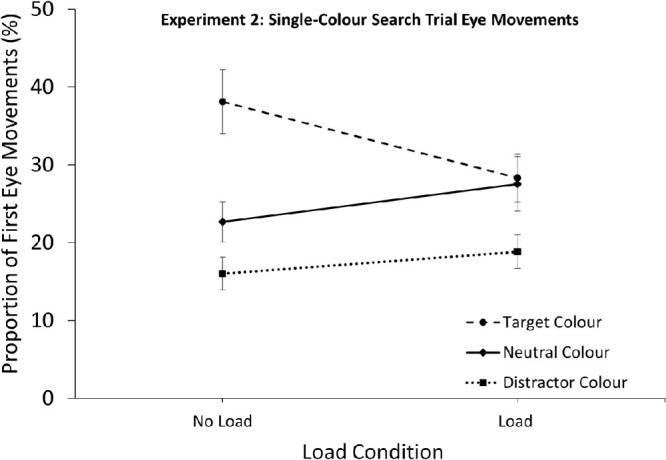
Proportions of first eye movements directed toward each of the three singleton types in the single-color trials. Results revealed that in both load and no-load conditions, the distractor-colored singleton attracted fewer first eye movements than the neutral-color singleton. The target color singleton strongly attracted eye movements, compared to the neutral color under no load, but this effect was completely eliminated under load. *Error bars* represent within-subject 95% CIs (Loftus & Masson, 1994).

The RT result were complimented by the first eye movements directed toward the differently colored singletons. A 3 (Singleton Color: Target, Distractor, Neutral) × 2 (Load: No load, Load) repeated-measures ANOVA was conducted on the proportions of first eye movements landing on the different colors (see Figure 6). There was an effect of singleton color, *F*(2, 66) = 36.33, *p* < 0.001, ƞ^2^_p_ = 0.52, no effect of memory load, *F*(1, 33) = 0.15, *p* = 0.706, but, importantly these effects were qualified by a significant interaction, *F*(2, 66) = 9.05, *p* < 0.001, ƞ^2^_p_ = 0.22. The no load condition showed evidence for both target enhancement and distractor suppression: there was a significant effect of singleton color, *F*(2, 66) = 35.79, *p* < 0.001, ƞ^2^_p_ = 0.52, with the target-color singleton attracting more first eye movements than the neutral singleton, *t*(33) = 4.84, *p* < 0.001, 95% CI [9.0, 22.0], whereas the distractor-color singleton received fewer first eye movements than the neutral colors, *t*(33) = 3.30, *p* = .002, 95% CI [2.5, 10.7]. Importantly, target feature enhancement and distractor suppression behaved differently under load. Although there was still an effect of singleton color, *F*(2, 66) = 8.32, *p* < 0.001, ƞ^2^_p_ = 0.20, the target color no longer attracted more eye movements than the neutral colors under load, *t*(33) = 0.29, *p* = 0.771, BF_10_ = 0.19. To confirm, we included a Bayes factor analysis: A BF_10_ < 1 shows support for the null hypothesis (and < 0.3 indicates *very* strong evidence for the null; Quintana & Williams, 2018), thus this result indicated the attentional bias toward the target color was eliminated under load. The distractor-color singleton continued to attract less eye movements than the neutral singleton *t*(33) = 3.39, *p* = 0.002, 95% CI [3.5, 13.9] under load. To confirm that load did not impact distractor-color capture we compared proportions of first fixations for the distractor-color singleton between the two load conditions and found no differences *t*(33) = 1.49, *p* = 0.146, BF_10_ = 0.50. The neutral singleton also did not vary across load conditions; *t*(33) = 1.76, *p* = 0.088, BF_10_ = 0.73. These results closely matched the RT data of the single-color search trials (with the addition of an observable difference between distractor and neutral colors) and indicated that target-feature enhancement was eliminated with load, while distractor-feature suppression continued.

### Single-color search: Eye movements to the target-shape

To further examine search performance in the single-color search trials, the proportions of first fixations landing on the shape-target was analyzed. One participant was excluded from this analysis due to low rates of target-shape capture (<15%). A 2 (Load: No load, Load) × 3 (Singleton color: Target, Distractor, Neutral) repeated-measures ANOVA on first saccade proportions to the shape target was conducted. Means are displayed in Table 1. There were effects of memory load: *F*(1, 32) = 4.69, *p* = 0.038, ƞ^2^_p_ = 0.13, distractor color: *F*(2, 64) = 14.04, *p* < 0.001, ƞ^2^_p_ = 0.31, and an interaction: *F*(2, 64) = 10.34, *p* < 0.001, ƞ^2^_p_ = 0.24. The *t*-tests revealed that shape-target capture was reduced under load for when the singleton was either the distractor color: *t*(32) = 3.49, *p* = 0.001, 95% CI [3.2, 12.0], or a neutral color: *t*(32) = 3.14, *p* = 0.004, 95% CI [3.1, 14.4]. When the singleton was the color of the target from the visual search trials, proportions of first saccades toward the shape target actually *increased* under load: *t*(32) = −2.11, *p* = 0.043, 95% CI [−11.7, −0.2]. Like target capture rates in the visual search trials of Experiment 1, a general reduction in localization was observed under load (excluding the target-color singleton trials).

**Table 1.** Single-color search: Proportions of first saccade directed toward the shape-target

Singleton-color	No load	Load
Target	26.9%	32.8%
Distractor	41.4%	33.8%
Neutral	40.3%	31.6%

## Discussion

Experiment 2 investigated the differential impact of working memory load on two mechanisms that contribute to the facilitation effects observed in the presence of a distractor: target-feature enhancement and distractor-feature suppression. In the neutral single-color search trials attentional allocation to target and distractor colors were compared to a neutral color. With no VWM load both target enhancement and distractor suppression led to behavioral guidance in eye movement data (the RT data was not sensitive enough to distinguish between the neutral and distractor singletons). The target-colored singleton attracted first eye-movements more so than the neutral singleton, whereas the distractor-colored singleton attracted less. These results seem to describe how attention was guided in the visual search trials. Both enhancing to the target-color and suppressing the distractor-color, would lead attention to be biased toward the targets and non-targets more so than the distractor.

Interestingly, the addition of VWM load differentially impacted behaviors toward the target and distractor singletons. For the distractor-colored singleton the addition of load did not influence behavior. There was no increase in distractor-color fixations with the addition of load, and first fixations were still lower than the neutral colors. This suggests that distractor-feature suppression does not rely on working memory resources to guide attention. Conversely, the target-color singleton was dramatically impacted with the addition of load. Under load, there were no longer any observable biases toward the target-color compared to neutrals. The target-color singleton no longer led to longer RTs, and had the same proportion as first fixations as the neutral singletons. This indicated that the non-defining aspect of the target (its color) no longer contributed to attentional guidance, suggesting that the representation bias toward the (irrelevant) target color in part relied on working memory resources.

The elimination of target-enhancement but not distractor-suppression has implications for the observations in the visual search trials. Under no VWM load, the inhibition of the distractor was guided by two mechanisms, attending to the target color and suppressing the distractor color. In the single-color search trials, these mechanisms were independently measured, the inhibition of the distractor-singleton could only have been driven by suppression, as target-colors were not present in the display. When adding load to the tasks, there was no effect on the distractor color in the single search trials (as the only guidance process was suppression), whereas in the visual search trials the target-enhancement was attenuated, leaving only suppression to guide attention away from the distractor. This would account for the reduced suppression effects observed under load in the visual search trials.

These results were complimented by the search efficiency for the shape target in the single-color search trials. When memory load was added to the task, shape-target capture increased for target-colored singleton, indicating that search performance improved. Conversely, shape-target capture was reduced for distractor and neutral colored singleton trials under memory load. This result seems to depict the overall impact of VWM load on feature guided search. Capture rates for the neutral and distractor singletons did not increase, yet the localization of the shape-target was less efficient under load. This seems to indicate that adding VWM led to poorer search performance, but importantly that this decrease in target localizations was not related to increases in singleton capture.

## General discussion

The current experiments tested the role of VWM in static-feature inhibition. As outlined above, arguments have been made that distractor inhibition could be based on a working memory template for rejection (e.g., Arita et al., 2013; Sawaki & Luck, 2010). Inhibition could also arise as a result of automatic feature priming or selection history effects, which would not requiring working memory resources (Kristjánsson & Driver, 2008; Maljkovic & Nakayama, 1994). Both accounts predict that in visual search, exposure to a distractor would lead attention to become biased away from irrelevant features. Although both explanations provide the same functional prediction, the distinction between the two is important for understanding the interplay between the automatic and strategic mechanisms of the visual system.

The results of the two experiments revealed that distractor-feature suppression can operate independently from VWM. When introducing a concurrent memory load into visual search, the distractor was still suppressed and continued to guide attentional selection, facilitating target selection. In Experiment 1 we observed an RT facilitation effect in the presence of a distractor, which has been the trademark for inhibition in previous visual search studies (e.g. Gaspelin et al., 2015; Gaspelin et al., 2017). In addition, first eye movements were less likely to select the distractor than one of the non-target items, and although inhibition was reduced under load, there were no observable changes across low to high memory loads in Experiment 1. Although the continuation of inhibition under load provides evidence against a rejection-based template, the weakening of the suppression effect in the eye movement data warranted further investigation.

Experiment 2 distinguished between the influences of target-feature enhancement and distractor-feature suppression under load. Previous studies have revealed that both, the prioritization of target (and non-target) features and the inhibition of distractor feature contribute to RT facilitation on distractor-present trials (Chang & Egeth, 2019; Hamblin-Frohman et al., *submitted*). With no memory load, these finding were replicated. In the single-color search trials the target colored singleton attracted attention more so than a neutral color, while the distractor color attracted attention less than the neutral color. For distractor suppression, this effect was observed to the same extent under memory load as without. That attention displayed the same bias away from the distractor singleton (compared to neutral singleton) suggests that this bias was disconnected from VWM. Target-feature enhancement appears to be the result of different mechanisms than distractor-feature suppression. With VWM load, the capture effects observed in the no load condition (slower RTs and higher proportion of first eye movements) were completely eliminated, and attentional selection of the target color singleton was no different to the neutral colors. This implies the target-color attentional biases that helped to guide search behavior was dependent on VWM resources.

A common standpoint in the visual search literature is that the top-down goals of the searcher are likely to be stored in working memory (Desimone & Duncan, 1995; Folk et al., 1992; Wolfe, 2021). It appears that the top-down goal used in visual search did not just incorporate the defining shape of the target, but also its non-distinguishing color; i.e. participants searched for the “blue diamond,” not just the “diamond”. Several studies have shown that selective attention and stored memory items can compete for working memory resources (Hamblin-Frohman & Becker, 2019; Kiyonaga & Egner, 2014). In line with this view, the addition of memory load in the current design appears to have significantly diminished the influence of the top-down representation, so that the task-irrelevant color of the target was no longer encoded in memory, or prioritized for selection. Conversely, the preservation of distractor-color suppression under load suggests that this guidance was not template-based.

One unresolved issue of the current data is the increase in distractor fixations under VWM load in both experiments 1 and 2. In the visual search trials there were both reductions in target fixations and decreases in distractor fixation when memory load was added to the task. Importantly, there was no accompanying increase in non-target capture rates, which seems to suggest that first fixations were diverted away from the target to the distractor. At face value this result appears to suggest that VWM load attenuated inhibition of the distractor feature. However, in the single color search trials there was no such increase in distractor-color fixations across load conditions. The key difference between these the visual and single-color search trials was the presence of the target-color in the non-target items. As described above, the attentional bias toward the (non-defining) target color was eliminated under VWM load. Thus under VWM load there was lower bias toward the non-target items, rendering the distractor color as relatively more salient under load compared to without. The key is that both distractor-feature suppression and target-feature enhancement were used to guide search *away* from the distractor. With added VWM load this process was likely reduced to just distractor-feature suppression, accounting for the reduction in the oculomotor suppression effect.

If inhibition is not due to a working memory template does this mean that it is necessarily due to a selection history effect? Awh et al. (2012) argue that selection history effects should be considered an independent process, as recent interactions with a stimulus feature can cause changes in attentional selection that cannot be accounted for by top-down or bottom-up mechanisms. This is certainly true for the distractor used in this study. In the single-color trials, the colored singleton would be considered as extremely salient compared to the other stimuli (it was the only item with a deviant color). A stimulus-driven account of attention would predict that all of the singletons should capture attention due to the high feature contrast of the colored item compared to the colorless non-target items (Theeuwes, 1992). Thus the reduction in capture for the distractor colored singleton (compared to the neutral) cannot be accounted for by salience-based capture. In the current data we do not have the evidence to claim that selection history effects are responsible for the observed feature inhibition; however, this explanation currently remains as the most plausible.

Selection history effects have been described as a form of implicit memory, which operates independently of the explicit working and short-term memory (Wiggs & Martin, 1998). Several studies have drawn distinctions between priming and other memory forms: single-cell recording reveal differential activations for repeated stimuli compared to goal-related stimuli (Miller & Desimone, 1994), priming effects occur in the absence of conscious memory (in amnesiac patients; Hamann & Squire, 1997) and attentional interference only impacts encoding of explicit memory contents, not implicit memory contents (Szymanski & MacLeod, 1996). The effects of repeated item exposure appear to exhibit *contingent automaticity* (Bargh, 1989), because they are largely automatic, do not require explicit instructions but at the same time depend on top-down goals and task demands (e.g., in that priming effects are typically stronger for task-relevant than irrelevant features; see Becker, 2007; Becker, 2008a; Becker, 2008b; Becker, 2008c; Becker, 2010b; Fecteau, 2007). These studies suggest a distinction between implicit priming and explicit working memory. The inhibition effects in the current study appear to follow the predicted patterns of implicit memory processes.

One point of concern is that only the distractor color seemed to be influenced by repeated exposure, whereas the target color did not. Classically, repetition priming studies have noted that the influence of feature repetition is present for both target and distractor features (Kristjánsson & Driver, 2008; Lamy, Antebi, Aviani, & Carmel, 2008; Maljkovic & Nakayama, 1994). However, in the single-color search trials, under memory load, the target colored distractor did not attract attention more than the neutral color, suggesting that no selection history effects had occurred for the target color. Furthermore, Carlisle and Kristjánsson (2018) examined interactions between VWM and inter-trial priming. They varied whether or not VWM contents overlapped with visual search targets and distractors. They found that inter-trial priming continued to facilitate search RTs when there was either no relationship between VWM contents and the search items, or when the repeated target color was retained in VWM. When the distractor color was retained (including when it was retained along with the target color) no inter-trial priming was observed. The target and distractor retention effects both concur with our data. A target item that is retained in VWM forms the attentional template for search, leading to facilitations in search behavior. Conversely, when the distractor was forced into a template by the VWM task, there was conflict between memory and priming signals. Interestingly, the authors observed priming (for both target and distractor) when there was no overlap with VWM, which at face value contradicts the lack of target priming observed in the single-color search trials of Experiment 2.

It is surprising that the repetition of the distractor color led to a persistent suppression effect, whereas consistent repetition of the target color did not lead to an enhancement effect under load. In the current experiment, the defining search-related feature of the target was its shape (diamond) and its color (e.g., blue) was associated, but task-irrelevant (because it was shared with non-targets). Priming studies have noted that repetition effects are stronger for the critical, target-defining feature of an item rather than associated task-irrelevant features (Becker, 2008c; Fecteau, 2007). In the single-color search trials, the singleton only contained the (non-defining) color information, and not the (target-defining) shape. This differed from the design of Carlisle and Kristjánsson (2018) as their study revealed repitition priming effects of the *task-relevant* feaure while under memory load. It seems that in the current design with a VWM load, priming did not extend to the irrelevant target-color. Conversely, the distractor was defined only by its color in both visual search and single-color trials, thus allowing inhibitory priming to divert attention away from the feature under load.

One further potential consideration is that the search could have been guided by activated long-term memory (a-LTM) systems. A-LTM theories posit that repeated information does not necessarily require storage in WM but can be accessed from a long-term store when needed (Oberauer, 2009). The advantage of this is that a large amount of relevant information can be stored, which only needs to be activated when the need arises from corresponding task demands (D'Esposito & Postle, 2015). In the current design, participants completed 40 no-load practice visual search trials that included distractor-present trials. When a repeated search target is used, its representation can rapidly transition from working memory into long-term memory (Carlisle, Arita, Pardo & Woodman, 2011); however, further research has suggested that this is only possible for a single item (Berggren & Eimer, 2017). Although the actual storage of the search stimuli could conceivably be in a-LTM, it does not account for why VWM impacted the ability to search for the target in both experiments. VWM load reduced the ability to locate the target stimulus in all conditions. Of specific interest is the shape-target capture on the single-color search trials, which decreased when load was added to the neutral or distractor-singleton present trials. Importantly the reduction in target-capture was not accompanied by an increase in singleton-capture, suggesting that the cause was a weakening of the representation of the target item (or the ability to search for the target), not a reduction in ability to ignore singletons. Conversely, in the target-color singleton trials, singleton capture was dramatically reduced under load. Together, both shape and color feature information of the target item was reduced or eliminated under VWM load, suggesting that even if these representations were stored in a-LTM, VWM was still needed to guide attention.

## Conclusions

Although the current study has not definitively shown that inhibition is due to a selection history effect, it has provided strong evidence against template-based rejection. Distractor-feature suppression in visual search has been demonstrated to continue to guide attention, even under the influence of memory load. This implies that suppression is a mechanism that can operate independently of templates stored in working memory. It is still entirely possible that under specific experimental paradigms, rejection-based templates may be able to be used. However, as soon as trial *n + 1* of a repeated distractor feature (e.g., Gaspelin et al., 2019), suppression via a selection history or priming mechanism is likely to contribute to observed behavioral inhibition.

## Supplementary Material

Supplement 1
