# Supplementary material for: Inhibition continues to guide search under concurrent visual working memory load
Source: J Vis. 2022 Feb 14;22(2):8. doi: 10.1167/jov.22.2.8 (PMC8857620; doi:10.1167/jov.22.2.8)
Supplement: Supplement 1 [file jovi-22-2-8_s001.pdf]

Appendix.

Memory accuracy and inhibition effects

Analyses were re-run on some of the main results from Experiments 1 and 2, however, only including data from trials in which the memory task was accurately responded to. Potentially trials with incorrect responses may not have reflected the true interaction between memory and search due to the memory trial being prioritised lower. We could not make comparisons between correct memory trials and incorrect memory trials as the trial numbers were too low for incorrect memory responses (~33% for the high load condition, ~15% for the low load).

Table 1.

Visual Search: RTs on correct-only memory trials.

	No Load (all trials)	Low Load	High Load
**Experiment 1**			
Distractor Absent	943.8ms	985.4ms	978.6ms
Distractor Present	892.1ms	929.0ms	954.6ms
**Experiment 2**			
Distractor Absent	958.1ms	971.6ms	-
Distractor Present	920.5ms	939.8ms	-

Experiment 1: A 2(Distractor: Present, Absent) x 3(Load: No load, Low load, High load) repeated measures ANOVA was conducted on the mean RTs in the visual search trials. A significant effect of distractor presence was observed, $F(1, 24) = 38.96$, $p < .001$, $\eta^2_p = 0.62$, with RTs being faster on distractor present trials compared to absent trials. There was a significant effect of memory load $F(2, 48) = 5.73$, $p = .006$, $\eta^2_p = 0.19$, with the no load condition being faster than both low: $p = .024$ and high loads: $p = .013$. There was, however, a significant interaction differing from the main analysis, $F(2, 48) = 3.88$, $p = .027$, $\eta^2_p = 0.14$. The difference arose in the high load condition where the distractor facilitation effect just failed to reach significance, $t(24) = 2.03$, $p = .053$. The most likely explanation for this was due to the lack of

reliability from the reduced trial numbers in the high load condition (33% memory errors, compared to 15% in the low load).

Experiment 2: a 2(Distractor: Present, Absent) x 2(Load: No load, Load) repeated measures ANOVA was conducted on the mean RTs in the visual search trials. There was no effect of load $F(1, 33) = 2.39$, $p = .132$. A significant effect of distractor presence appeared $F(1, 33) = 36.73$, $p < .001$, $\eta^2_p = 0.53$ indicating that RTs were faster when the distractor was present compared to absent. There was no interaction indicating that this effect was consistent across load condition, $F(1, 33) = 0.33$, $p = .569$. These results were consistent to the main analysis.

Relationship between memory performance and inhibition effect in visual search

Correlations were performed between memory accuracy for each load condition and the RT inhibition effect in the visual search trials (measured as distractor absent – distractor present trials). For both load condition there were no significant relationships. Low: $r(25) = .18$, $p = .377$, and high, $r(25) = .13$, $p = .545$. This was the same in the load condition of Experiment 2: $r(34) = .20$ $p = .254$. These results did not suggest any relationship between the memory capacity of the individual participants and their ability to use inhibition to facilitate search.

Table 2. First fixation proportions on distractor-present trials (correct-only memory)

	No Load (all trials)	Low Load	High Load
**Experiment 1**			
Target	44.30%	37.68%	36.69%
Non-Target	22.46%	22.05%	22.65%
Distractor	9.50%	17.60%	17.71%
**Experiment 2**			
Target	45.09%	39.44%	-
Non-Target	22.45%	21.08%	-
Distractor	10.01%	18.01%	-

These values were almost identical to the inclusive data set of correct and incorrect memory responses and thus the analyses was omitted for brevity. In summary; examining the

data using correct-only memory trials did not meaningfully change the interpretations of the full analysis.

Further eye-movement analyses

The following analyses investigate some of the eye-movement mechanics that were not assessed in the main body of text. An analysis of distractor and non-target dwell times was planned, however die to the limited trials where participants fixated on the distractor the data was not sufficient enough to be analysed.

Table 3.

Visual Search: Target localisation time (time for first gaze fixation upon target)

	No Load	Low Load	High Load
**Experiment 1**			
Distractor Absent	429.4ms	502.7ms	513.9ms
Distractor Present	383.2ms	452.8ms	483.2ms
**Experiment 2**			
Distractor Absent	440.6ms	477.9ms	-
Distractor Present	417.3ms	449.6ms	-

Experiment 1: A 2(Distractor: Present, Absent) x 2(Load: No load, Low load, High load) repeated measures ANOVA was conducted on the mean target localisation times. A significant effect of distractor presence was observed, $F(1, 24) = 29.43$, $p < .001$, $\eta^2_p = 0.55$, with RTs being faster on distractor present trials compared to absent trials. There was a significant effect of memory load $F(2, 48) = 36.47$, $p < .001$, $\eta^2_p = 0.60$, with the no load condition being faster than both low: $p < .001$ and high loads: $p < .001$. There was no difference between low and high loads: $p = .070$. There was no interaction $F(2, 48) = 0.12$, $p = .886$.

Experiment 2: A 2(Distractor: Present, Absent) x 2(Load: No load, Load) repeated measures ANOVA was conducted on the mean target localisation times. A significant effect of distractor presence was observed, $F(1, 33) = 31.73$, $p < .001$, $\eta^2_p = 0.49$, with localisation being faster on distractor present trials compared to absent trials. There was a significant effect of

memory load $F(1, 33) = 22.62$, $p < .001$, $\eta^2_p = 0.41$, with the target being located quicker under the no load condition. There was no interaction $F(1, 33) = 0.27$, $p = .609$.

The results from the target localisations were consistent across both Experiments 1 and 2. The presence of the distractor in visual search led to quicker localisations of the target item under all load conditions, and appear to account for the RT facilitation effects reported in the main body of the text (magnitude of the differences were roughly equivalent in all conditions). A general interference of memory load was also observed, with the addition of the memory task leading to slower target localisations.

Table 4.

Visual Search: First Saccade Latency

	No Load	Low Load	High Load
**Experiment 1**			
Distractor Absent	209.0ms	215.7ms	202.2ms
Distractor Present	208.0ms	216.7ms	227.3ms
**Experiment 2**			
Distractor Absent	218.6ms	213.7ms	-
Distractor Present	221.7ms	216.2ms	-

First saccades were counted as the first eye movement that landed outside the fixation region within a time frame of 100ms – 500ms.

Experiment 1: A 2(Distractor: Present, Absent) x 3(Load: No load, Low load, High load) repeated measures ANOVA on first saccade latencies was conducted. There were no effects of distractor presence: $F(1, 24) = 2.11$, $p = .160$, memory load: $F(2, 48) = 1.08$, $p = .349$, or an interaction $F(2, 48) = 2.72$, $p = .076$.

Experiment 2: A 2(Distractor: Present, Absent) x 2(Load: No load, Load) repeated measures ANOVA on first saccade latencies was conducted. There was no effects of memory load: $F(1, 33) = 2.10$, $p = .156$, or an interaction: $F(1, 33) = 0.93$, $p = .076$. Surprisingly the effect of distractor presence returned significant: $F(1, 33) = 6.11$, $p = .019$, $\eta^2_p = 0.16$, with first

saccades being slightly delayed (~3ms) in distractor present trials. It is hard to interpret this effect due to low magnitude of the difference as well as it not being observed in Experiment 1.
